# Frequency of Congenital Cardiac Disease in Various Divisions of Khyber Pakhtunkhwa (KPK) Based on a Six-Month Analysis of Inpatients at a Tertiary Care Hospital’s Pediatric Cardiology Unit

**DOI:** 10.7759/cureus.35209

**Published:** 2023-02-20

**Authors:** Farman Ali, Haseen Dil Wazir, Ali Akbar, Abdul Moeed Khan, Yasir Rehman, Ijaz Hussain, Huma Gul, Ikram Ullah, Khadim Khan, Aamir K Afridi

**Affiliations:** 1 Pediatric Cardiology, Peshawar Institute of Cardiology, Peshawar, PAK; 2 Pediatric Cardiology, Lady Reading Hospital, Peshawar, PAK; 3 Pediatrics, Peshawar Institute of Cardiology, Peshawar, PAK

**Keywords:** pediatric cardiology, children, divisions, khyber pakhtunkhwa, congenital cardiac disease

## Abstract

Objective: To identify the frequency of congenital heart disease (CHD) in various Khyber Pakhtunkhwa (KPK) divisions.

Materials and methods: To perform this research, we reviewed the medical records of pediatric cardiology patients hospitalized between January 2022 and July 2022. Data was taken from the cardiology department's computerized data system between January 1 and July 31, 2022. To prevent any errors in diagnosis and address them during input by the ward clerk, patients' addresses, diagnoses, and gender were verified with the computerized medical record. Data were analyzed, and frequency and percentages were calculated. Per the digital mapping of KPK districts, we stratified and examined the data among various KPK divisions.

Results: Out Of the 371 patients with CHD who were admitted, 36.98% (137) were from the Peshawar division, 28.84% (107) from the Malakand division, 16.71% (62) from the Mardan division, 6.1% (23) from the Kohat division, 4.3% (16) from the Bannu division, 3.5% (13) from the Dera Ismail Khan division, and 3.5% (13) from the Hazara division. A total of 371 had CHD, of which 234 (63.07%) were cyanotic and 137 (36.92%) were cyanotic. The most prevalent acynotic congenital heart defect was patent ductus arteriosus (PDA) at 36.32% (85), followed by a ventricular septal defect (VSD) at 35.04 (82). The most frequent acyanotic congenital cardiac abnormality was tetralogy of Fallot (TOF) at 49.63% (68), which was followed by transposition of the great arteries (TGA) at 33.57% (46).

Conclusion: In KPK, congenital heart disease is most prevalent in the divisions of Peshawar and Malakand, while it is least prevalent in Hazara.

## Introduction

Congenital heart defects (CHD) are characterized as structural and functional cardiac deformities that, if not treated with the proper procedures, may result in a person's reduced quality of life and even early death [1]. Nine-tenths of the world's congenital heart disease patients reside in areas with poor treatment and a high death rate [2]. The incidence of CHDs varies significantly by ethnic group, particularly for certain abnormalities associated with high infant mortality. Healthcare services must reduce racial disparities, especially via early CHD detection during screening, parental decision support, and efficient therapies. Future studies should examine the causes of ethnic variance and how they affect longer-term results [3]. Agadoorappa et al. conducted a study to look into ethnicity variation by reviewing a large cohort of babies at birth and found that those children whose parents belong to Pakistan have more CHDs as compared to others [4]. There are eight to 10 instances of congenital cardiac disease for every 1000 live births. White people have a considerably greater prevalence of CHD than Black or Mexican Americans do [5]. Van Der Linde et al. discovered substantial regional disparities after conducting a comprehensive evaluation; 9.3 per 1,000 live births was the CHD newborn prevalence rate in Asia [6]. State-specific variations account for around 180,000 CHD births in India each year, which accounts for 10% of the country's current infant mortality [7]. The prevalence of CHD in Sudan is 2.0 per 1000, which is similar to rates in other African nations but lower than those in Europe and North America [8].

Children with CHDs have different types of presentation in the emergency department (ED): they may present in shock or cardiac failure or for murmur evaluation for the first time, or they may come to the emergency for management of spells or chest infection, or they may have a problem after cardiac surgery. Therefore, every child who presents in critical condition at the ED should have a suspicion of heart disease and be evaluated thoroughly for CHD as recommended by Bano et al. [9]. Outside of infancy, tetralogy of Fallot (TOF) is the most prevalent cyanotic cardiac condition. One-tenth of all congenital cardiac disease is caused by TOF [10]. The most prevalent cyanotic lesion is TOF, while the noncyanotic lesion is ventricular septal defect (VSD) [11].

The lack of diagnostic facilities and access to healthcare in many regions of the globe contributes to the disparities in reported birth prevalence between high and low-income nations. Further research is required to customize the treatment of the global health issue of CHDs given that observed disparities may also be of genetic, environmental, socioeconomic, or ethnic origin [4]. Although there have been a few hospital-based studies undertaken at the regional level to demonstrate the prevalence of CHD, statistics on incidence or prevalence at the national level that may demonstrate the burden of CHD in Pakistan are sadly lacking [12]. To our knowledge, there is currently no information on the prevalence of congenital cardiac disease in the various KPK areas.

Our research's objective is to determine the prevalence of congenital cardiac disease throughout various KPK divisions. This will assist us in the future to focus our health resources on a particular demographic and geographic region while also allowing us to explore any disparities that we see that may have genetic, environmental, socioeconomic, or ethnic roots.

## Materials and methods

Study design

Patients hospitalized at the pediatric department of the Peshawar Institute of Cardiology (PIC) were recruited in this descriptive and prospective hospital-based research study

Inclusion and exclusion criteria

All patients from KPK with CHD admitted at the pediatric unit of our hospital were included in the study. All patients with acquired heart disease, syndromic babies with congenital heart disease, premature babies with patent ductus arteriosus or patients from other provinces were excluded from the study.

Data collection 

Our intention was to utilize a well-designed data sheet to gather data for at least six months of our anticipated study. Between January 1 and July 31, 2022, data were extracted from the computerized data system of the cardiology department. Their residence, illness, and gender were checked against the computerized medical record to make sure there were no mistakes made when the ward clerk entered their information. We filtered and looked at the data among different KPK divisions in line with digital mapping of KPK districts. The essential data on the form was filled up using information from interviews with study participants and data from their hospital records. By including the hospital registration number, data duplication was avoided. Overall, 371 individuals were found, and data sheets were finished.

Statistical analysis 

The collected data were entered and analyzed. Frequencies, means, and percentages are all components of calculated descriptive statistics.

Ethical consideration

The ethical committee of Peshawar Institute of Cardiology granted approval for this study on 12/12/2022 (approval no. HEC/12/12/194).

## Results

In our study, there were a total of 371 patients out of which 201 (55%) patients were female and 170 (45%) were male (Table 1).

**Table 1:** Gender-wise distribution

Gender	Number (%)
Male	170 (45%)
Female	201 (55%)

Acyanotic CHD was diagnosed in 234 (63.07%) patients while cyanotic CHD was diagnosed in 137 (36.92%) (Table 2).

**Table 2:** Lesion-wise distribution of CHD CHD: Congenital heart disease

Type of CHD	Number (%)
Acynotic	234(63.07%)
Cyanotic	137(36.92%)

According to our data, CHD is more common in the Peshawar division (36.98%) followed by the Malakand division (28.84%), Mardan division (16.71%), Kohat division (6.1%), Bannu division (4.3%), Hazara division (3.5%), and Dera Ismail Khan division (3.5%). Of the 371 patients, 137 (36.98%) were from Peshawar. Out of these 137 patients, 78 (56.93%) had acyanotic congenital heart abnormalities, whereas 59 (43.06%) had cyanotic ones. The most frequent acyanotic CHD was patent ductus arteriosus (PDA) followed by VSD, while the most frequent cyanotic CHD was TOF followed by transposition of the great arteries (TGA). Of the total number of patients, 107 (28.84%) were from the Malakand division, and among these patients, 78 (72.89%) had acyanotic CHD, and 29 (27.10%) had cyanotic CHD. The most frequent acyanotic CHD was VSD followed by PDA, while the most frequent cyanotic CHD was TOF followed by TGA and pulmonary atresia (PA). Out of 371 patients overall, 62 (16.71%) were from the Mardan division where 50% were cyanotic and 50 % were acyanotic. In this division of KPK, VSD was the most common acyanotic CHD followed by PDA, while the most prevalent cyanotic CHD was TGA followed by TOF. Twenty-three (6.19%) out of the 371 patients came from the Kohat division. Twenty (86.95%) of these patients were acyanotic, while three (13.04%) were cyanotic. The most frequent cyanotic CHD was TOF, whereas the most frequent acyanotic CHD was VSD followed by PDA. From the Bannu division, there were 16 patients (4.31 %). Six (37.5%) of them were cyanotic, whereas 10 (62.5%) were acyanotic. The most frequent acyanotic CHD in this division was PDA followed by VSD, while the most frequent cyanotic CHD was TOF followed by TGA and PA. From the Dera Ismail Khan division, there were 12 patients (3.2%). Among them, nine (75%) were acyanotic, and three (25%) were cyanotic. The most frequent acyanotic CHD here was PDA followed by VSD, while the most frequent cyanotic CHD was TOF followed by TGA. Twelve of the 371 total patients were from the Hazara division. In this group of 12 patients, four (33.33%) were cyanotic and eight (66.66%) were acyanotic. In this division of KPK, PDA was the most prevalent acyanotic CHD followed by VSD, while TGA was the most prevalent cyanotic CHD followed by TOF and PA (Tables 3, 4).

**Table 3:** Division-wise distribution of acyanotic CHD PDA: Patent ductus arteriosus, VSD: Ventricular septal defect, ASD: Atrial septal defect, PS: Pulmonary stenosis, AS: Aortic stenosis, CAVSD: Complete atrioventricular septal defect, COA: Coarctation of aorta, CHD: Congenital heart disease

Division	PDA	VSD	ASD	PS	AS	CAVSD	C0A
Peshawar	32	20	16	03	04	03	00
Malakand	21	33	13	03	02	04	02
Mardan	11	15	02	02	01	00	00
Kohat	07	08	02	02	00	01	00
Bannu	05	02	01	01	00		01
Hazara	05	01	00	01	00	01	00
Dera Ismail khan	04	03	01	01	00	01	00
Total	85	82	35	13	07	09	03

**Table 4:** Division-wise distribution of cyanotic congenital heart disease TOF: Tetralogy of Fallot, TGA: Transposition of great arteries, PA: Pulmonary atresia, TA: Tricuspid atresia, MA: Mitral atresia, TAPVCP Total anomalous pulmonary venous connection

Division	TOF	TGA	PA	TA	MA	TAPVC
Peshawar	32	17	05	02	00	03
Malakand	15	05	05	05	01	00
Mardan	11	20	00	00	00	00
Kohat	03	00	00	00	00	00
Bannu	04	01	01	00	00	00
Hazara	01	02	01	01	00	00
Dera Ismail khan	02	01	00	00	00	00
Total	68	46	12	07	01	03

## Discussion

According to Mitchell et al., CHD is described as a gross anatomical anomaly of the heart or intrathoracic major vessels that is actually or potentially functionally significant. This description does not include persistent left superior vena cava or the coupled brachiocephalic-left carotid arterial trunk, which are both functionless anomalies of the great veins [13].

Our study showed acyanotic CHD in 234 (63.07%) cases while cyanotic CHD was found in 137 (36.92%) cases making a ratio of 1:1.7, which means acyanotic is more common than cyanotic lesions. Similar results were obtained in a study conducted by Nicholas et al. in a tertiary referral center [1]. 

Around the globe, 0.8% of newborns are born with congenital cardiac disease [14]. The bulk of these congenital abnormalities has an unknown cause, however, genetic factors are increasingly understood to play a more significant influence [15]. According to our study results, congenital cardiac disease is more common in females 201 (55%) as compared to males 170 (45%). Interestingly regional and global studies have demonstrated opposite results where boys are more affected than girls [7,3,11].

Historically, early childhood was when most CHD patients passed away. However, this field of medicine has made amazing progress during the last four decades. With a consistent rise in age at death and a decline in mortality, death from CHD has shifted towards adults than from newborns [16]. About 250,000 individuals in the UK are CHD patients whose condition is referred to as grown-up CHD, and this number is rising. Most of these patients, about 50% of them, are females of reproductive age. For women with CHD, the stress of pregnancy creates a new impute [15].

In 2017, it was predicted that there were almost 1.8 occurrences of CHD per 100 live births worldwide [2]. The frequency of CHD at birth is believed to vary across areas and nations owing to variations in genetic, environmental, and epigenetic factors [17]. According to Sadiq et al., Asian newborns had a higher estimated prevalence of CHD than non-Asian infants (9.45 per 1000 vs. 4.56 per 1000, p=0.0001) [18]. Heart diseases that are present at birth and may be diagnosed later by a cardiologist make up a large bulk of cardiovascular disease as investigated by Rehan et al. in a study conducted in KPK; they found that 16.76 % of patients referred to the cardiac diagnostic center of cardiovascular disease were having CHD, with VSD being the most common lesion followed by ASD [19].

According to a study by Zahid et al., acyanotic CHD (52.8%) is more prevalent than cyanotic CHD (47.1%), which is similar to our study. However, their study did not examine the range of CHDs or the relative distribution of CHD in the various divisions of KPK [20]. Our research showed that there are significant geographical differences in KPK regarding the occurrence of CHD as it is more common in urban and populated divisions such as Peshawar and Malakand. Our data showed regional variation in the prevalence of different types of lesions both acyanotic and cyanotic. Overall PDA (36.32%) was the most common acaynotic lesion followed by VSD (35%). Among cyanotic lesions, TOF (49.63%) was the most common lesion followed by TGA (33.57%). In a study conducted in India, an almost similar pattern was detected which stated that TOF was the most common cyanotic lesion while VSD was the most common acyanotic heart defect [8].

Our research is distinctive since it is the first local study to concentrate on regional variations in CHD. And this will aid and direct us in the future towards more extensive research to help identify the many etiological and environmental elements contributing to CHD. According to our findings, CHD is more prevalent in urban regions than in non-urban areas.

Our study has few limitations: it is a single-centered study, and patients seen in other centers across the province were not included. The results might have been different if data was collected at the division level as the patients might be referred to other nearby centers. 

## Conclusions

According to our data, in KPK, congenital cardiac disease is more prevalent in the Peshawar and Malakand divisions, while it is least prevalent in the Hazara division. Acyanotic CHD is more common in KPK as compared to cyanotic CHD. Acyanotic PDA was the most common defect followed by VSD, while TOF was the most common lesion followed by TGA in the cyanotic group. Congenital heart disease is more prevalent in females as compared to males according to our study. 
